# Chemical Constituents, In Silico Studies and In Vitro Antioxidant, Enzyme Inhibitory and Antibacterial Activities of the Algerian *Tamarix boveana* Essential Oil and Extracts

**DOI:** 10.3390/plants14162497

**Published:** 2025-08-11

**Authors:** Esma Lamera, Imad Mennai, Nabila Slougui, Chawki Bensouici, Hichem Hazmoune, Houssem Boulebd, Salah Akkal, Gema Nieto

**Affiliations:** 1 Chemistry Department, Faculty of Exact Sciences, Brothers Mentouri University Constantine 1, Constantine 25000, Algeria; lamera.esma@umc.edu.dz (E.L.); mennai_imad@umc.edu.dz (I.M.); 2 Unit for the Valorisation of Natural Resources, Bioactive Molecules and Analyses Physicochemical and Biological Analyzes (VARENBIOMOL), Department of Chemistry, Faculty of Science, University Metouri-Constantine 1, Constantine 25000, Algeria; hichemhazmoune@yahoo.fr (H.H.); salah4dz@yahoo.fr (S.A.); 3 Laboratory of Process Engineering for Sustainable Development and Health Products, National Polytechnic School of Constantine, Ville Universitaire Ali Mendjli, Constantine 25016, Algeria; slougui.nabila@gmail.com; 4 National Center For Biotechnology Research, Ali Mendjli, Nouvelle Ville UV 03, Constantine BP 25011, Algeria; c.bensouici@crbt.dz; 5 Laboratory of Synthesis of Molecules with Biological Interest, Frères Mentouri Constantine 1 University, Constantine 25017, Algeria; boulebd.houssem@umc.edu.dz; 6 Department of Food Technology, Food Science and Nutrition, Faculty of Veterinary Sciences, Regional Campus of International Excellence Campus Mare Nostrum, University of Murcia, Espinardo, 30071 Murcia, Spain

**Keywords:** GC–MS, in silico, BuChE, *α*-amylase, in vitro, DFT calculations, molecular docking

## Abstract

The objective of this study is to evaluate, for the first time, the chemical composition and the antioxidant, enzyme inhibitory, photoprotective and antibacterial properties of the *Tamarix boveana* essential oil (EO) as well as its organic extracts. The analysis of the EO obtained from the aerial parts of *T. boveana* was carried out employing the technique of gas chromatography with flame ionization detection (GC-FID) and mass spectrometry (GC-MS). Forty-four constituents were identified, constituting 91.18% of the oil, with the major compounds being *γ*-cadinene (9.41%), *β*-caryophyllene (6.71%), limonene (6.5%), *p*-cymene (6.16%), copaene (4.37%), terpinen-4-ol (4.23%), *δ*-cadinene (4.21%) and *γ*-terpinene (4.11%). The antioxidant activity of *T. boveana* essential oil and organic extracts (hydroalcoholic, CHCl_3_, AcOEt, *n*-BuOH) was evaluated by different tests, including DPPH, ABTS, phenanthroline, SNP and ferric reducing power. The findings indicated that *T. boveana* essential oil possesses moderate antioxidant capacity, with IC_50_ values of 223.59 ± 1.01 μg/mL according to the DPPH test. The extracts and essential oil also demonstrated notable inhibitory impacts against *α*-amylase and butyrylcholinesterase. Antimicrobial activity was determined regarding four bacterial strains, determining the minimum inhibitory concentrations (MICs) and bactericidal concentrations (MBCs). The geometry and electronic properties of the main EO compounds were determined using density functional theory (DFT) calculations. Furthermore, docking studies were conducted to investigate the interaction and binding affinity of these molecules with the active sites of BuChE and *α*-amylase enzymes. The results highlight the value of *Tamarix boveana* as a medicinal plant and indicate its effectiveness as an important source of bioactive compounds for many uses.

## 1. Introduction

Since ancient times, humans have used nature, mainly plants, to develop remedies for a variety of diseases. Thus, natural compounds have been the foundation of drug development over the decade [1]. Phytochemicals possessing biological properties, such as antioxidants, antimicrobials, antimutagens and anti-inflammatories, are extensively utilized to treat various human disorders, including Alzheimer’s disease (AD) and diabetes mellitus [2].

Essential oils are natural volatile secondary metabolites obtained from aromatic plants, recognized for their unique smells and diverse biological effects [3]. Owing to their prospective health advantages and natural sources, these oils are utilized across multiple sectors, including medicine [4], food [5] and cosmetics [6]. Essential oils are increasingly employed in food preservation due to their capacity to suppress microbiological proliferation, hence prolonging shelf life and maintaining food safety [7]. Pharmaceutically, they exhibit promise as natural therapies for infections, with studies demonstrating their efficacy against multidrug-resistant bacteria, thereby establishing them as significant contenders in combating antibiotic resistance [8]. To this fact, the production of essential oils from plants and the assessment of their efficacy remain in high demand due to their significant worth.

Polyphenols present in plants have been recognized for their diverse pharmacological effects, including antioxidant, antibacterial, anticancer, antihypertensive and anti-inflammatory actions [9,10,11]. These compounds are essential in the prevention of oxidative stress caused by the excessive generation of free radicals that can harm biological components. This damage causes aging and the development of several diseases associated with age, including cancer, cardiovascular disease and degenerative disorders.

In addition, polyphenols can provide skin protection against UV rays by absorbing and reducing the generated free radicals [12].

Butyrylcholinesterase and *α*-amylase are essential targets for drugs and extracts because their inhibition is involved in the management of Alzheimer’s disease (AD) and diabetes mellitus (DM) [13,14]. Cholinesterase inhibitors, such as galantamine, are used for AD, while acarbose regulates glucose levels in diabetes mellitus. However, these treatments have side effects, such as hepatotoxicity and gastrointestinal disorders [15,16,17]. Therefore, there is a necessity for new natural inhibitors without unwanted side effects to treat these two diseases. These alternatives could offer safer and more effective solutions.

The genus *Tamarix,* of the family Tamaricaceae, includes approximately 104 species taxonomically characterized and accepted [18]. These species are known to thrive in saline–alkaline soils as they tolerate harsh abiotic conditions, including high temperatures, salinity and drought [19,20]. *Tamarix* species are distributed in Asia, North Africa, North America and Europe. In traditional medicine, *Tamarix* species are used to treat various diseases [21,22]. For instance, *T. gallica* has demonstrated a range of biological activities, including anti-Alzheimer’s, anticancer, antidiabetic and antibacterial properties [18]. As well, *T. articulata* has been used as a therapeutic option against several diseases for decades; this plant has shown antibacterial, antiviral, anticancer, antioxidant and anti-inflammatory properties [22,23,24]. Other species, like *T. aphylla*, provide antimicrobial, antioxidant and anti-inflammatory properties [25,26]. In particular, *T. boveana* is well recognized for its antibacterial and insecticidal activities [18,27,28].

Numerous phytochemical investigations conducted on different *Tamarix* species have shown the presence of several active compounds, notably polyphenols [21]. On the other hand, no studies have been conducted on essential oils and organic extracts from Algerian *T. boveana*. As far as we know, there are no reports on the phytochemical composition or studies on the biological activity of this species, especially regarding enzyme inhibition and photoprotective properties. This paucity of scientific information reveals the novelty and interest of the present study. To this end, efforts will be made to include a unified assessment of such aspects for the first time. As part of ongoing research on Algerian medicinal plants [29,30,31], GC/MS and GC-FID analyses were used to determine the chemical composition of the essential oil of *T. boveana* aerial parts. This study also intended to evaluate the antioxidant, enzyme inhibitory, photoprotective and antimicrobial activities of the essential oil as well as the hydroalcoholic, CHCl_3_, AcOEt and *n*-BuOH extracts of the aerial parts of *T. boveana*. This polarity gradient approach was intended to maximize the chemical diversity of the extracts and enable correlation between individual extract types and their respective observed biological activities.

## 2. Results and Discussion

### 2.1. T. boveana Essential Oil Chemical Composition

**Figure 1:**
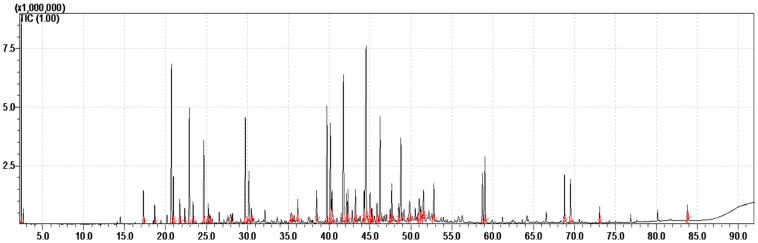
GC-MS Chromatogram of the EO obtained from Algerian *T. boveana* (the integrated and identified peaks are in red).

The essential oil of the whole aerial parts of the species *T. boveana* is obtained by steam distillation, yielding 0.33 ± 0.01% (*w*/*w*) relative to the dry plant matter. It is a yellowish oil with an aromatic smell. The results of GC-FID and GC/MS analyses of essential oil are presented in Table 1. Furthermore, the chromatogram presented in Figure 1 illustrates the abundance of secondary metabolites in the essential oil.

A total of 44 compounds were identified in the whole aerial part of *T. boveana*, representing 91.18% of the volatile compounds. The major constituents of the essential oil are *γ*-Cadinene (9.41%), *β*-caryophyllene (6.71%), Limonene (6.5%), *p*-Cymene (6.16%), Copaene (4.37%), Terpinen-4-ol (4.23%), *δ*-Cadinene (4.21%) and *γ*-Terpinene (4.11%) (Table 1).

The predominant chemical components were observed to be sesquiterpene hydrocarbons (37.9%), followed by a significant amount of monoterpene hydrocarbons (26.01%). Additionally, a small amount of oxygenated sesquiterpenes (9.47%) and oxygenated monoterpenes (9.15%) were identified (Table 1).

The chemical profile of *T. boveana* essential oil, harvested in Algeria, is reported here for the first time. This allows for a comparative study with the same species harvested in other regions or with other *Tamarix* species whose essential oil chemical composition has been reported. For instance, Saïdana et al. [27] published GC-FID and GC/MS analysis of the essential oil from the whole aerial parts of *T. boveana* harvested in Tunisia. Initially, it is apparent that the essential oil of Tunisian *T. boveana* is richer in secondary metabolites containing 62 compounds (93.32% of the total oil), dominated by hexadecanoic acid (18.14%), docosane (13.34%), germacrene D (7.68%) and fenchyl acetate (7.34%). The Tunisian essential oil is abundant in fatty acids, fatty acid esters and hydrocarbons, while the Algerian *T. boveana* essential oil is characterized by a higher terpene content. Unique compounds like Edusma-4 (15),7-diene-1-*β*-ol and cis-Muurola-4 (14),5-diene are found in the Algerian *T. boveana* essential oil. In contrast, 2.4-Nonadienal is specific to Tunisian oil.

On the other hand, Alhourani et al. [32] analyzed the GC and GC-MS profiles of the essential oil from the aerial parts of *T. aphylla* (L.). At first sight, *T. boveana* essential oil has more identified compounds than *T. aphylla* (33 identified compounds representing 89.75% of the total oil). *T. aphylla* oil is dominated by non-terpenoid non-aromatic hydrocarbons (52.39%), with 6,10,14-trimethyl-2-pentadecanone (32.39%) as the predominant component, followed by *β*-ionone (13.74%) and dodecanoic acid (6.00%). *T. boveana* oil shows a higher fraction of sesquiterpenes (47.7%) compared to monoterpenes (35.78%), while *T. aphylla* oil is rich in oxygenated sesquiterpenes (26.53%).

Unique compounds like Edusma-4 (15),7-diene-1-*β*-ol and cis-Muurola-4 (14),5-diene are found in *T. boveana* oil, with *γ*-Cadinene as the major component (9.41%), which is not reported in *T. aphylla* oil. Conversely, *T. aphylla* contains a small amount of *α*-thujone (0.57%), which is not mentioned in our study. This dissimilarity can be explained by variances in extraction methodology, along with variations in geographical conditions and the fact that it is a distinct species [33,34,35].

Several of the major compounds identified in *Tamarix boveana* essential oil, such as β-caryophyllene [36], Terpinen-4-ol [37] and γ-cadinene, are known for their strong antioxidant, anti-inflammatory and antimicrobial activities [38]. β-Caryophyllene has demonstrated significant antioxidant [36] and anti-inflammatory properties [39], while Terpinen-4-ol contributes to microbial inhibition and wound healing [40]. Although less studied, γ-cadinene has shown notable antioxidant activity in the essential oils of other plant species [41]. These bioactivities likely explain, at least in part, the in vitro and in silico effects observed in this study. The synergistic action of these volatile compounds enhances this oil’s pharmacological potential.

### 2.2. In Vitro Antioxidant Ability

Plant antioxidants can inhibit or prevent reactive oxygen species (ROSs), acting as a regulator of antioxidant defense, thus protecting the human body from oxidative stress. In this study, the antioxidant capacity of the essential oil and the different extracts of *T. boveana* was evaluated and compared to many references (Table 2). The antioxidant capacity of the essential oil and extracts was evaluated by five methods: DPPH radical scavenging, ABTS, Phenanthroline, Silver Nanoparticles SNP and the reducing power assay.

Table 2 indicates that the *n*-BuOH extract has the most significant antioxidant activity (*p* < 0.05) in DPPH radical scavenging (IC_50_ = 47.45 ± 4.82 μg/mL), although this value is lower than that of the reference substances (BHT, BHA, *α*-tocopherol, tannic acid and ascorbic acid, Table 2). The hydroalcoholic extract exhibited an IC_50_ of 64.16 ± 1.77 μg/mL, followed by the AcOEt extract (IC_50_ = 91.34 ± 0.65 μg/mL), the CHCl_3_ extract (IC_50_ = 131.68 ± 0.01 μg/mL) and, finally, the essential oil (IC_50_ = 223.59 ± 1.01 μg/mL), which showed the lowest antioxidant activity. The essential oil’s low antioxidant activity may be due to its limited content of polyphenolic and flavonoid compounds, which are generally responsible for radical-scavenging mechanisms and are more prevalent in polar extracts. The other tests, ABTS, reducing power, Phenanthroline and SNP assays, also confirmed the antioxidant potential of the *n*-BuOH extract, which showed higher activity than the hydroalcoholic extract, succeeded by the AcOEt extract, the CHCl_3_ extract and, finally, the essential oil, which showed the lowest activity in all tests (Table 2, *p* < 0.05). Furthermore, in some tests, the *n*-BuOH extract demonstrated superior antioxidant capacity compared to reference antioxidants. In the reducing power test, the *n*-BuOH extract (IC_50_ = 23.42 ± 0.5 μg/mL) showed better activity than BHT (IC_50_ = 152.24 ± 2.43 μg/mL, *p* < 0.05), tannic acid (IC_50_ = 41.07 ± 2.36 μg/mL, *p* < 0.05) and *α*-tocopherol (IC_50_ = 34.93 ± 2.38 μg/mL, *p* < 0.01). Similarly, for the SNP assay, the *n*-BuOH extract (IC_50_ = 32.21 ± 0.73 μg/mL) showed higher activity than α-tocopherol (IC_50_ = 34.93 ± 2.38 μg/mL, *p* < 0.001), used as the standard.

A correlation study was conducted among DPPH, ABTS, Phenanthroline, SNP and the reducing power tests of *n*-BuOH extract (Table 3). Table 3 reveals highly positive linear correlations (*R* > 0.88) among all tests, particularly between the reducing power, DPPH and ABTS assays. These correlations indicate that these tests have comparable predictive capabilities to assess the antioxidant activities of *n*-BuOH extract.

The total phenol and flavonoid contents (Table 4) showed that the species *T. boveana* is a significant source of phenolic compounds, particularly the *n*-BuOH extract, which exhibits the highest concentrations of total phenols and flavonoids (563.70 ± 3.40 μg AGE/mg and 124.79 ± 0.26 μg QE/mg, respectively, *p* < 0.05) compared to the hydroalcoholic, AcOEt and CHCl_3_ extracts.

Based on previous research, the *n*-butanol extract was found to be more effective for retrieving phenolic compounds [42]. Indeed, the *n*-butanolic extract demonstrated the best antioxidant activity, primarily due to its polarity. This characteristic improves the efficient extraction of phenolic compounds and flavonoids, which are known for their antioxidant properties [43].

**Figure 2:**
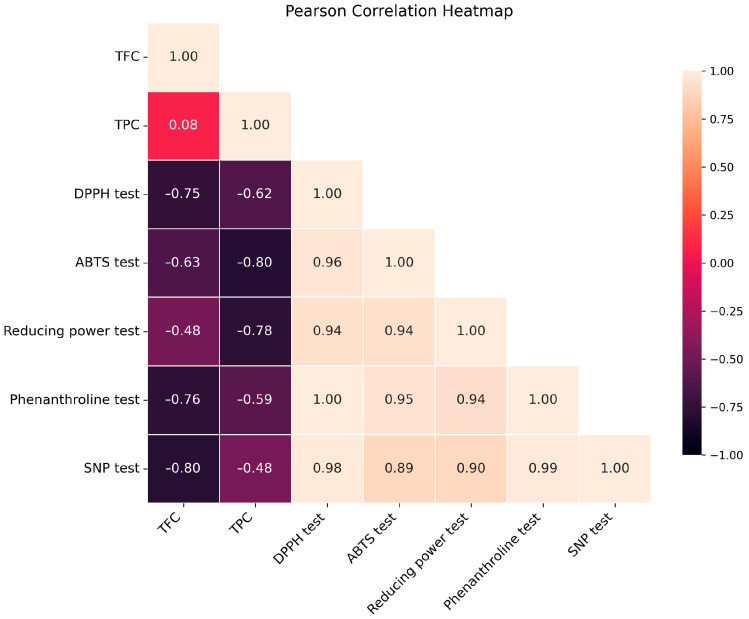
Pearson’s correlation heatmap between antioxidant parameters and TPC/TFC of *n*-BuOH extract.

A Pearson correlation analysis was conducted to examine the correlations between antioxidant assay results and phenolic/flavonoid concentrations (Figure 2). The heatmap demonstrated significant negative associations between TPC/TFC and IC_50_ or A_0_._5_ values, indicating that increased phenolic/flavonoid content correlates with enhanced antioxidant activity. The *n*-BuOH extract, demonstrating the highest total phenolic content (TPC) and total flavonoid content (TFC), displayed the most significant antioxidant effects in all experiments. This corroborates the concept that antioxidant activity is predominantly influenced by the concentration of phenolic and flavonoid compounds [44,45]. These results are presented as preliminary indicators of polyphenolic and flavonoid content, intended to support a statistically grounded correlation with the observed biological activities, rather than as definitive evidence of chemical composition.

Through our study, the antioxidant activity of extracts and essential oil of the Algerian *T. boveana* was evaluated for the first time. However, some antioxidant tests (DPPH, ABTS) on *T. boveana* extracts harvested in Tunisia have already been confirmed by Saidana Naija et al. [46], who reported that ethyl acetate and methanolic fractions exhibited DPPH IC_50_ values close to 80 µg/mL. In contrast, our *n*-BuOH and hydroalcoholic extracts demonstrated superior antioxidant potential, with significantly lower IC_50_ values. Additionally, while the Tunisian study focused on a limited number of radical-scavenging assays, our antioxidant assessment was based on five complementary tests (DPPH, ABTS, Phenanthroline, SNP and reducing power), allowing for a more comprehensive evaluation of antioxidant mechanisms.

### 2.3. Enzyme Inhibition Effects

**Figure 3:**
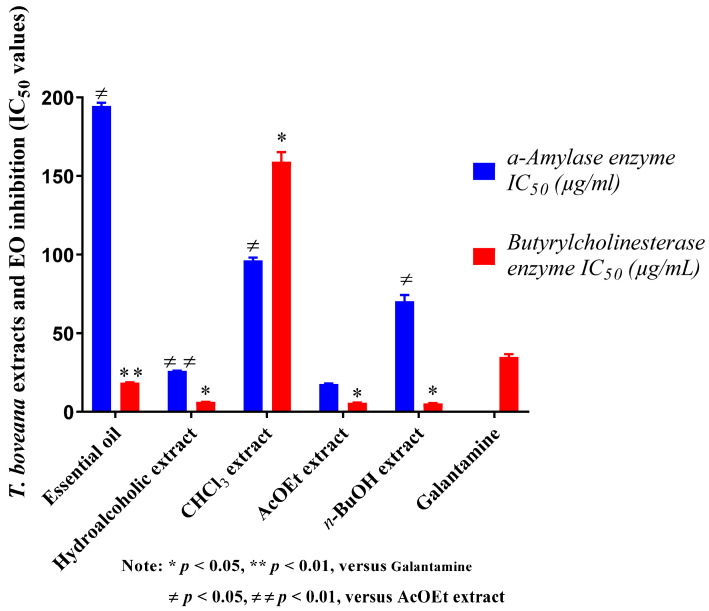
The results of *T. boveana* extracts and EO inhibition (IC_50_ values) against *α*-Amylase and BuChE enzymes.

Inhibition of butyrylcholinesterase is considered an effective means to treat Alzheimer’s disease (AD) [47]. Therefore, the essential oil and all extracts of *T. boveana* were evaluated for their ability to inhibit BChE. The results are provided in Figure 3 with IC_50_ in μg/mL. The samples demonstrated dose-dependent inhibitory effects against BChE. *n*-BuOH, AcOEt and hydroalcoholic extracts exhibited extreme BChE inhibitory effects with an IC_50_ of 5.44 ± 0.40 µg/mL, 5.92 ± 0.40 μg/mL and 6.44 ± 0.06 μg/mL, respectively, in contrast to the standard Galantamine (IC_50_ = 34.75 ± 1.99 μg/mL, *p* < 0.05) (Figure 3). The essential oil also demonstrated significant activity against BChE (IC_50_ = 18.35 ± 0.96 μg/mL), unlike the standard Galantamine (*p* < 0.01). The CHCl_3_ extract (IC_50_ = 159.17 ± 1.74 μg/mL, *p* < 0.05) showed the lowest activity. The anticholinesterase activity of *T. boveana* essential oil was evidently associated with its high concentrations of sesquiterpenes and monoterpenes, especially *γ*-Cadinene (9.41%), *β*-caryophyllene (6.71%), *α*-pinene (2.59%) and *β*-Phellandrene (1.12%), which have been suggested for their cholinesterase inhibitory activities [48,49].

The *α*-amylase assay indicates that our extracts and essential oil exert dose-dependent antidiabetic activity. They were proven to have measurable inhibitory activity against the involved enzyme in diabetic disease. The results indicate that the AcOEt extract exhibited the most significant inhibitory activity against *α*-amylase (IC_50_ = 17.56 ± 1.38 μg/mL) followed by the hydroalcoholic extract (IC_50_ = 25.92 ± 0.18 μg/mL, *p* < 0.01), the *n*-BuOH extract (IC_50_ = 70.43 ± 4.38 μg/mL, *p* < 0.05) and, lastly, the CHCl_3_ extract (IC_50_ = 96.71 ± 1.66 μg/mL, *p* < 0.05). The essential oil exhibited the lowest activity with an IC_50_ of 194.67 ± 7.92 μg/mL, but this value remains higher than that of acarbose, which was used as a reference (IC_50_ = 3650.93 ± 10.70 μg/mL).

The activity of *T. boveana* essential oil against *α*-amylase is perhaps due to the presence of monoterpene and sesquiterpene molecules, including *β*-caryophyllene (6.71%), *α*-pinene (2.59%) and *p*-Cymene (6.16%), which are known for their α-amylase inhibitory activity [50,51].

Through this work, we present the first study concerning the enzymatic inhibitory activity of extracts and essential oil of *Tamarix boveana,* harvested in Algeria, against *α*-amylase and butyrylcholinesterase. To our knowledge, no assessment of α-amylase and BuChE inhibition exists yet for T. boveana grown in other regions. However, a literature search revealed that several species of the genus *Tamarix* have been assessed for their *α*-amylase and cholinesterase inhibitory activities, and the obtained results are positive [52].

### 2.4. Photoprotective Activity

The sun protection factor (SPF) of a sunscreen shows the protection degree offered by this product against UV-B radiation and measures the skin’s protection against sunburn. This factor is determined in the laboratory through standardized tests [53].

An effective sunscreen should cover a wide absorption range, from 290 to 400 nm. This study aims to evaluate for the first time in vitro the SPF of essential oil and extracts of *T. boveana*. The protection factors against UV-B radiation are summarized in Table 5. According to this table, the *n*-BuOH, hydroalcoholic and CHCl_3_ extracts presented the highest SPF values (46.40 ± 0.00, 46.10 ± 0.03 and 41.28 ± 0.3, respectively) compared to standards, including Vichy sunscreen (SPF = 44.22 ± 0.1) and Nivea sunscreen (SPF = 50.11 ± 0.22). In contrast, the essential oil showed the lowest SPF value (SPF = 9.08 ± 1.18, *p* < 0.05).

The SPF values of the extracts appear to be directly related to their polyphenol content. Indeed, *T. boveana* extracts showed high SPF values, probably due to their richness in polyphenols, which can absorb UV radiation in the 280–320 nm wavelength range [54]. This UV absorption capacity makes these extracts promising candidates for developing potent photoprotective agents.

### 2.5. Antibacterial Activity

The antibacterial potential of *T. boveana* essential oil and extracts was confirmed by the appearance of a bacterial growth inhibition zone. The activity was determined by measuring minimum inhibitory concentrations (MICs) and minimum bactericidal concentrations (MBCs). The microbial resistance of *Staphylococcus aureus*, *Pseudomonas aeruginosa*, *Escherichia coli* and *Micrococcus luteus* strains is presented in Table 6. The essential oil, as well as all extracts, showed inhibition of microbial growth, depending on the strains’ sensitivity and the sample concentration, as shown in Table 6.

The results revealed that the essential oil (EO) effectively inhibited the growth of all tested strains, with MIC values between 1.5 and 3 μg/mL and MBC values between 4 and 8 μg/mL, which are similar to those of gentamicin (MIC: 2 μg/mL, MBC: 6 μg/mL, *p* < 0.01). The EO mainly showed high efficacy against *Micrococcus luteus*, with respective MIC and MBC values equal to 1.5 μg/mL and 4 μg/mL, for *p* < 0.01. However, the *n*-BuOH and hydroalcoholic extracts showed similar antibacterial efficacy against *Micrococcus luteus*, with MIC and MBC of 3 and 10 μg/mL, respectively, which are higher than CHCl_3_ and AcOEt extracts, for *p* < 0.01. Regarding *Staphylococcus aureus*, the CHCl_3_ extract showed more marked antibacterial activity, with an MIC of 3 μg/mL and MBC of 5 μg/mL, compared to the hydroalcoholic, *n*-BuOH and AcOEt extracts.

This study reports, for the first time, the antimicrobial properties of the essential oil and extracts of *T. boveana* harvested in Algeria. However, other research has documented similar antibacterial activities of the essential oil from *T. boveana* harvested in Tunisia [27].

The antimicrobial efficacy of *T. boveana* essential oil appears to be consistent with the presence of *β*-caryophyllene, a major component (6.71%) in this essential oil. This compound has shown in vitro activity against *S. aureus*, *E. coli* and *P. aeruginosa*. Furthermore, volatile compounds, such as *β*-caryophyllene, might be responsible for the noted antimicrobial properties, potentially related to their role in forming resins or complex menthols, which are also said to possess antibacterial characteristics [27].

### 2.6. Density Functional Theory Calculation

**Figure 4:**
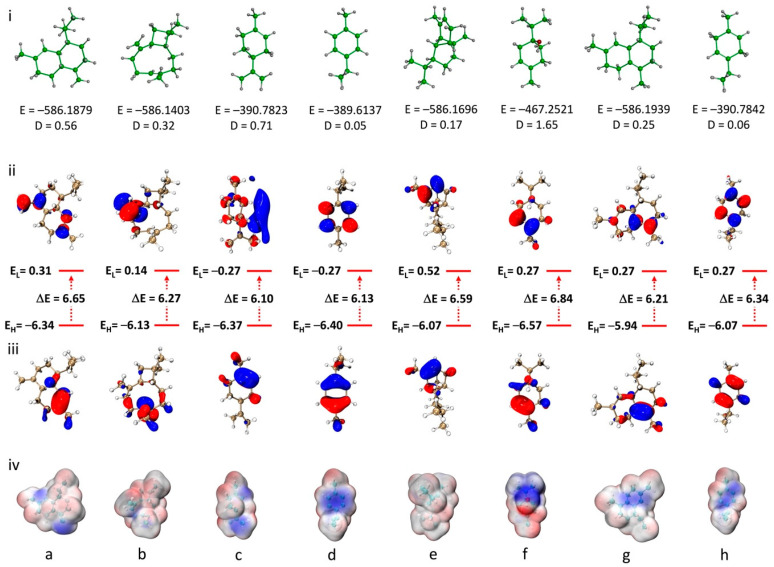
(**i**) Computed molecular geometry; (**ii**) LUMO; (**iii**) HOMO; and (**iv**) ESP of: (**a**) *γ*-cadinene; (**b**) *β*-caryophyllene; (**c**) limonene; (**d**) *p*-cymene; (**e**) copaene; (**f**) terpinen-4-ol; (**g**) *δ*-cadinene; and (**h**) *γ*-terpinene at B3LYP/6-311G (d,p) level.

Quantum chemical calculations were performed for the main EO compounds to better understand their geometry, electronic properties and chemical reactivity. All calculations were performed using the DFT method at the B3LYP/6-311G (d,p) theoretical level. Analysis of the molecular geometry (Figure 4i) reveals variations in total energy and dipole moments, indicating differences in stability and polarity that may influence the chemical properties and reactivity of the molecules. Terpinen-4-ol, with the highest dipole moment (D = 1.65 Debye), shows a pronounced polar character, suggesting a greater tendency to interact with polar environments or molecular systems. In contrast, compounds such as copaene and *γ*-terpinene, with lower dipole moments (D = 0.17 and 0.06 Debye, respectively), reflect a more non-polar, hydrophobic nature.

The frontier orbitals, HOMO (highest-occupied molecular orbital) and LUMO (lowest-occupied molecular orbital), play a crucial role in the chemical reactivity of organic compounds. For the studied compounds, as shown in Figure 4ii and iii, the energy gaps (ΔE) between HOMO and LUMO range from 6.10 to 6.84 eV, reflecting the electronic stability and potential reactivity of the molecules. For example, limonene and *p*-cymene, with moderate ΔE values (6.10 and 6.13 eV, respectively), suggest a good balance between chemical stability and reactivity compared to the other compounds. On the other hand, terpinen-4-ol, with a higher ΔE (6.84 eV), is associated with lower chemical reactivity due to its enhanced stability. The HOMO energy levels of the studied compounds, which reflect their ability to donate electrons and thus their antioxidant potential via the electron transfer mechanism, range from −5.94 to −6.57 eV. δ-cadinene shows the highest HOMO level (−5.94 eV), while terpinen-4-ol displays the lowest value (−6.57 eV), indicating that δ-cadinene may be the most potent antioxidant among the studied molecules. However, all HOMO values are lower than those of standard antioxidants such as Trolox (−5.39 eV) and BHT (−5.74 eV), which may explain the low antioxidant activity of the essential oil observed in the experimental studies [55].

Electrostatic potential (ESP) maps provide valuable insights into the distribution of electronic charges on the surface of molecules, highlighting regions likely to interact through electrostatic, nucleophilic or electrophilic interactions. The ESP maps of the studied compounds show varied charge distributions (Figure 4iv). For example, strongly negative regions (in blue) are observed around the terpinen-4-ol oxygen atom and double bonds, indicating potential sites for nucleophilic interactions. Conversely, slightly positive regions (in red), mainly observed on hydrogen atoms, correspond to sites likely to interact with nucleophilic species. Copaene and *δ*-cadinene, for example, show ESP distributions favoring hydrophobic interactions, while terpinen-4-ol, with a more heterogeneous distribution, suggests a higher polarity, implying the possibility of forming polar interactions. The Cartesian coordinates of the eight principal components of the calculated *Tamarix boveana* EO are reported in Table S1.

### 2.7. Docking Studies

EO has demonstrated significant inhibitory activity in vitro, corroborating its potential as a natural source of BuChE inhibitors. To elucidate the molecular basis of this activity, docking studies were carried out on the eight main components of EO towards the BuChE active site (PDB ID: 4bds). Docking analysis showed that most of the compounds exhibited favorable binding affinities, as reflected by their docking scores (Table 7). 

Among the studied molecules, *γ*-cadinene and *δ*-cadinene displayed the highest binding scores (−7.6 kcal/mol), suggesting their predominant role in the observed inhibitory activity. These compounds interacted primarily with TRP82 and HSD438 via Pi–alkyl and Pi–sigma interactions, highlighting the importance of hydrophobic interactions in stabilizing these molecules within the BuChE active site. Other significant contributors include *β*-caryophyllene and copaene, with docking scores of −7.5 kcal/mol and −7.3 kcal/mol, respectively. *β*-caryophyllene formed multiple interactions, including Pi–sigma and Pi–alkyl interactions, involving key residues such as TRP82, TYR332 and ALA328. Similarly, copaene exhibits interactions with TRP82, TYR332 and HSD438.

Compounds with moderate binding affinity, such as limonene, *p*-cymene, terpinen-4-ol and *γ*-terpinene, also demonstrated notable interactions with the BuChE active site. These interactions mainly involved Pi–alkyl and Pi–sigma contacts with residues such as TRP82, HSD438, ALA328 and TYR440.

**Figure 5:**
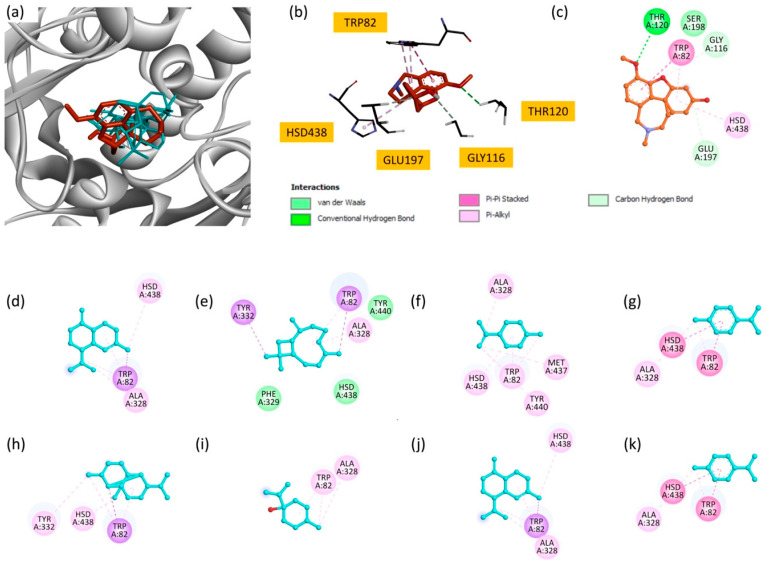
(**a**) Superimposition of all compounds and the reference molecule at the active site of BuChE; (**b**) bonding modes of galantamine; and (**c**) 2D representations of the interactions of galantamine; (**d**) *γ*-cadinene; (**e**) *β*-caryophyllene; (**f**) limonene; (**g**) *p*-cymene; (**h**) copaene; (**i**) terpinen-4-ol; (**j**) *δ*-cadinene; and (**k**) *γ*-terpinene.

Superimposition of all compounds and the reference inhibitor, galantamine (Figure 5a), revealed overlapping binding modes, suggesting that EO components may act through mechanisms similar to known inhibitors. Two-dimensional interaction maps (Figure 5c–k) further illustrate the involvement of critical active site residues, in particular TRP82 and HSD438, which are essential for BuChE activity [56].

EO also showed promising results against the *α*-amylase enzyme in vitro, although it was less effective than the extracts. Consequently, we also examined the interaction of the oil’s main components with *α*-amylase (PDB ID: 4gqr) via molecular docking. Among the analyzed compounds, *γ*-cadinene and copaene showed the highest docking scores (−7.3 kcal/mol), suggesting their strong binding to the *α*-amylase active site (Table 8). *γ*-cadinene mainly formed Pi–alkyl interactions with critical residues, such as TRP58, TRP59 and HSD305. Similarly, copaene exhibited Pi–sigma and Pi–alkyl interactions involving TYR62, ALA198, LEU165 and LEU162. These results indicate the importance of hydrophobic interactions in stabilizing these molecules in the enzyme’s active site. *δ*-Cadinene (−7.0 kcal/mol) and *β*-caryophyllene (−6.9 kcal/mol) also made significant contributions. *δ*-Cadinene formed multiple interactions, including Pi–alkyl and Pi–sigma contacts, with residues TRP59, TRP58, TYR62 and HSE299, highlighting its ability to interact with key catalytic residues. *β*-caryophyllene, despite its slightly lower binding score, formed stabilizing interactions consistent with its moderate inhibitory potential. Moderate binding affinities were observed for *p*-cymene (−5.9 kcal/mol), *γ*-terpinene (−5.9 kcal/mol), limonene (−5.8 kcal/mol) and terpinen-4-ol (−5.1 kcal/mol). These compounds interact primarily with residues such as TYR62, HSE299, LEU165 and LEU162 via Pi–alkyl and Pi–sigma interactions.

**Figure 6:**
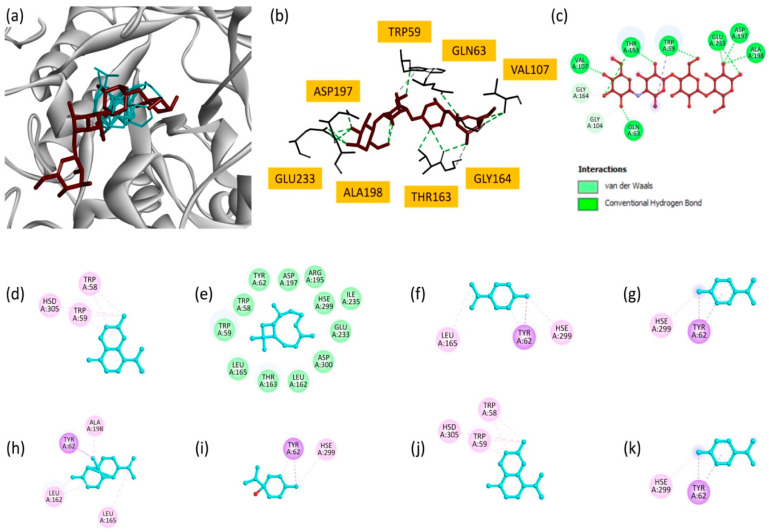
(**a**) Superimposition of all compounds and the reference molecule at the active site of *α*-amylase; (**b**) bonding modes of acarbose; and (**c**) 2D representations of the interactions of acarbose; (**d**) *γ*-cadinene; (**e**) *β*-caryophyllene; (**f**) limonene; (**g**) *p*-cymene; (**h**) copaene; (**i**) terpinen-4-ol; (**j**) *δ*-cadinene; and (**k**) *γ*-terpinene.

Superimposition of all docked compounds with the reference inhibitor, acarbose (Figure 6a), revealed overlapping binding modes, suggesting that EO components may share similar inhibitory mechanisms. Two-dimensional interaction maps (Figure 6d–k) highlighted the critical involvement of active site residues, in particular TRP58, TRP59, TYR62 and HSE299. These residues are essential for the enzyme’s catalytic function, and their interactions with EO components highlight the potential of these molecules to disrupt *α*-amylase activity [57].

In summary, docking studies revealed that the main components of the EO exhibit notable inhibitory potential against both BuChE and α-amylase, with favorable binding affinities and diverse interaction modes with the active sites of these enzymes. *γ*-cadinene and *δ*-cadinene were found to be the most potent inhibitors of BuChE (−7.6 kcal/mol), interacting mainly with TRP82 and HSD438 via hydrophobic interactions, while *γ*-cadinene and copaene showed the greatest affinity for *α*-amylase (−7.3 kcal/mol), forming Pi–alkyl and Pi–sigma interactions with residues such as TRP58, TYR62 and HSE299. Other constituents, such as *β*-caryophyllene and *p*-cymene, also demonstrated moderate activity towards both enzymes. The overlap in binding modes with the reference inhibitors suggests that the EO components may act via similar mechanisms, supporting their potential as natural inhibitors of both enzymes.

## 3. Conclusions

This study presents the first investigation on the chemical composition and biological properties of essential oil and extracts of *Tamarix boveana*, harvested in Algeria. The essential oil, which is rich in γ-cadinene, β-caryophyllene, limonene and p-cymene, exhibited noteworthy antimicrobial and enzyme inhibitory activities. The *n*-butanol extract, containing the highest concentrations of phenolic and flavonoid compounds, demonstrated strong antioxidant potential. Furthermore, docking studies revealed that the main components of the EO exhibit potential inhibitory activity against the BuChE and *α*-amylase enzymes, with notable binding affinities and various interaction modes.

This work offers significant new insights compared to previous studies, such as the Tunisian study conducted by Saïdana et al. [27]. It highlights a distinct chemical profile and provides a broader biological evaluation, including antioxidant activity, enzymatic inhibition (α-amylase and butyrylcholinesterase) and photoprotective potential. Additionally, this study is innovative in its integration of in silico methods, including DFT calculations and molecular docking, which simulate the interactions of the major compounds with the enzymatic active sites. These contributions considerably enrich existing knowledge, suggesting new potential therapeutic applications for this plant.

Although the results are promising, it is important to acknowledge certain limitations, such as the lack of in vivo validation and the absence of detailed toxicological data.

Further studies are warranted to confirm the biological activities through animal models and to investigate the action mechanisms of the active compounds. Additionally, broader safety and pharmacokinetic assessments will be required before potential therapeutic applications can be considered.

## 4. Materials and Methods

### 4.1. Plant Material

*T. boveana* aerial parts were collected in March 2022 at the flowering time from El-Bayadh region (33°40′49″ north, 1°01′13″ east) located in western Algeria in the steppe zone. The plant was identified by Pr. Mohamed Kaabache from Ferhat Abbas University in Setif. A sample specimen was placed in the herbarium of the research unit “VARENBIOMOL” in Constantine-1 University under the number TB/03/22. The plant material was dried for ten days in the shade at room temperature in the open air.

### 4.2. Essential Oil Extraction and Organic Extracts Preparation

Dried aerial parts of *T. boveana* (229 ± 4.96 g) were distilled for 3 h by steam distillation using a Kaiser Lang apparatus. The obtained essential oil was collected, extracted with hexane and then dried over anhydrous sodium sulfate (Na_2_SO_4_). The hexane was then allowed to evaporate at room temperature in open air. The resulting oil was stored at 4 °C for further analysis. The essential oil yield was calculated relative to the plant material weight, based on three replicates.

In addition, air-dried parts (697.1 ± 8.96 g) were cut into small pieces and macerated at room temperature in a MeOH–H_2_O (70:30 *v*/*v*) mixture at a rate of 1:40 (*w*/*v*) for 48 h, and this was repeated three times with solvent renewal. The filtrate was then concentrated and dissolved in water following filtration in order to obtain a hydroalcoholic extract (23.88 g) (yield was 3.43% ± 0.96). The resultant solution was sequentially extracted using organic solvents: CHCl_3_, AcOEt and *n*-butanol. The organic phases were dried using Na_2_SO_4_, filtered and then concentrated under vacuum, thus giving the different extracts as follows: chloroform (3.62 ± 0.96 g with a yield of 0.52 ± 0.02%), AcOEt (2.44 ± 0.46 g with a yield of 0.35 ± 0.03%) and *n*-butanol (15.6 ± 2.27 g with a yield of 2.24 ± 0.31%).

### 4.3. GC-FID Analysis

Quantitative analysis of the essential oil was determined using a Shimadzu gas chromatography (GC-FID) Model 2010, linked with a fused silica capillary column HP-5MS (30 m length × 0.25 mm ID. 0.25 *μ*m film thickness, 5%-diphenyl-95%-dimethylpolysiloxane), set to go from 50 °C (5 min) to 250 °C at 3 °/min and held for 10 min. The column was coupled to an injector (split mode 1/60) and a flame ionization detector (FID). The temperatures of the injector and the flame ionization detector were 280 and 300 °C, respectively. Acetone at a concentration of 3.5% *v*/*v* was used to dilute the essential oil. The used carrier gas was helium (1.0 mL/min).

Retention indices (RI) were determined using the Van den Dool and Kratz equation by analyzing standard alkanes (C_8_–C_20_) solutions under identical conditions.

### 4.4. GC/MS Analysis

GC-MS analysis of the EO was conducted using a Shimadzu gas chromatograph–mass spectrometer (model 7890/5975), combined with a capillary column HP-5MS (25 m length × 0.25 mm ID. 0.25 μm film thickness). The identical conditions program, referenced above in the GC-FID analysis section, was employed. MS quadrupole and ion source temperatures were 230 °C and 180 °C, respectively. The mass spectrometer was set to positive electron impact mode with an ionization voltage of 70 eV, and the electron multiplier was adjusted to 2200 V. Identification of the essential oil components was performed through comparing their retention indices (RI) and mass spectra with those of the reference compounds from the NIST 20 and Wiley 12 MS libraries. The relative proportions of each component were determined from the GC peak areas without the application of response factor correction; these percentages refer to the relative abundance of each compound based on the area under their respective peaks on the chromatogram (GC-MS), and not their absolute concentration in the essential oil. In other words, these values reflect the proportion of each compound in relation to the total peak area detected, rather than the actual mass or volume percentage in the oil [58].

### 4.5. Antioxidant Activity

#### 4.5.1. Total Flavonoid Content

Total flavonoid content was measured using the aluminum trichloride technique, with Quercetin as a reference component [59]. This approach involves generating a complex between flavonoids and aluminum, with a maximum absorption at 415 nm. The results are reported as µg equivalent of Quercetin per mg of extract (μg EQ/mg of extract).

#### 4.5.2. Total Polyphenol Content

Total phenolic content is usually evaluated colorimetrically with the Folin–Ciocalteu (FCR) assay using a 96-well microplate. The produced coloration is related to the amount of polyphenols present in the plant extracts determined spectrophotometrically [60]. Results are presented as µg gallic acid equivalent per milligram of extract (µg EAG/mg of extract).

#### 4.5.3. Scavenging Activity on DPPH Radical

The activity of extracts and EO to neutralize free radicals (DPPH) was evaluated using the method defined by Blois [61].

#### 4.5.4. ABTS Test

The ABTS scavenging activity of extracts and EO was evaluated at 734 nm, following the methodology described by Re et al. [62].

#### 4.5.5. Ferric Reducing Antioxidant Power

The reducing power of extracts and EO was evaluated using the Oyaizu [63] method with a slight modification.

#### 4.5.6. Phenanthroline Test

The reduction activity of the Phenanthroline complex [Fe (phen)_2_]^2+^ was determined by the method of Mansur et al. [64]. The results were set at A_0.50_ (μg/mL).

#### 4.5.7. Silver Nanoparticles SNP Activity

This activity is determined according to the method of Özyürek et al. [65]. The absorbance was recorded at 423 nm.

### 4.6. Photoprotective Activity (SPF)

Ultraviolet spectrophotometry serves as a supportive and preliminary in vitro approach for estimating the sun protection factor (SPF) of plant extracts and essential oils. The photoprotective effect was determined by the methodology of Mansur et al. [66,67]. The samples were put in ethanol at a concentration of 1 mg/mL (1000 ppm) and then ultrasonicated for 2 min to dissolve them and make a uniform solution. We used a multimode microplate reader (PerkinElmer Enspire, Singapore) to record absorbance across a range of 280–320 nm. We took three measurements for each and used the Mansur equation to figure out the SPF [53].

### 4.7. Enzyme Inhibitory Effect

#### 4.7.1. α-Amylase Inhibition

α-Amylase inhibitory activity was determined employing the method of Zengin et al. [68]. Acarbose is used as a standard.

#### 4.7.2. Cholinesterase Inhibition

The inhibitory activity of Butyrylcholinesterase (BChE) was determined by adopting Ellman’s technique [69], using galanthamine as a reference.

### 4.8. Antimicrobial Activity

The in vitro antimicrobial activity of extracts and essential oil was evaluated using the disk diffusion method [70]. Antimicrobial properties were tested against 4 bacterial strains, including Gram-positive bacteria (*Staphylococcus aureus* (ATCC 25923) and *Micrococcus luteus* strain (MM DSM 113600)) and Gram-negative bacteria (*Escherichia coli* (ATCC 25922) and *Pseudomonas aeruginosa* (ATCC 27853)).

#### MBC and MIC Determination

The minimum inhibitory concentration (MIC) and the minimum bactericidal concentration (MCB) were determined by applying the micro-dilution technique [71,72]. All analyses were repeated three times.

### 4.9. DFT Calculations

Quantum chemical calculations were conducted using Gaussian 09 software, employing the density functional theory (DFT) method at the B3LYP/6-311G (d,p) level [73]. Frequency calculations were performed to confirm that all compounds are in their ground states. Multiwfn 3.8 and VMD 1.9.3 software were used for the analysis and visualization of computational results [74,75,76]. Cartesian coordinates of the optimized structures for all compounds are provided in Table S1 of the Supporting Information.

### 4.10. Molecular Docking

The coordinates of the studied compounds were obtained from DFT calculations, while those of the proteins (human butyrylcholinesterase (BuChE) and human pancreatic *α*-amylase) were retrieved from the Protein Data Bank with identifiers 4bds and 4gqr, respectively. The proteins were prepared by removing ligands, water molecules, heteroatoms and co-crystallized solvents, followed by the addition of partial charges and hydrogens. The docking search space was defined as a 25 Å cube with grid points 1 Å apart, centered on the active site of the proteins. Docking studies were performed using AutoDock vina 1.1.2 software [77]. Figures were generated using BIOVIA Discovery Studio. The docking protocol was validated by comparing crystallographic and theoretical data for the native ligands, yielding RMSD values of 0.52 Å and 2.20 Å for 4bds and 4gqr, respectively.

### 4.11. Statistical Analysis

All calculated parameters were tested using the one-way analysis of variance (ANOVA). This analysis was repeated three times [78,79]. In case of statistical significance of the ANOVA test (*p* < 0.05), the differences in means between each treatment were examined using Tukey’s multiple comparison test.

## Figures and Tables

**Table 1:** Compounds identified from *T. boveana* essential oil.

No.	RT ^[a]^	RI_calc_^[b]^	RI_lit_^[c]^	Compunds ^[d]^	Relat. Conc ^[e]^
01	2.021	923	927	Tricyclene	1.38 ± 0.04
02	14.475	933	939	*α*-Pinene	2.59 ± 0.18
03	14.480	942	-	NI **^[f]^**	0.42 ± 0.02
04	17.301	964	1029	*β*-Phellandrene	1.12 ± 0.08
05	18.677	1018	1019	*α*-Terpinene	0.56 ± 0.02
06	20.131	1022	-	NI	0.82 ± 0.04
07	20.738	1025	1027	*p*-Cymene	6.16 ± 0.44
08	21.967	1030	1032	Limonene	6.50 ± 0.86
09	22.354	1046	1040	(Z)-*β*-Ocimene	0.46 ± 0.02
10	22.918	1058	1062	*γ*-Terpinene	4.11 ± 0.93
11	24.685	1087	1089	Terpinolene	3.13 ± 0.95
12	25.217	1102	1102	Linalool	0.62 ± 0.05
13	25.392	1122	1124	(Z)-*p*-Mentha-2,8-dien-1-ol	0.42 ± 0.09
14	26.612	1129	-	NI	1.50 ± 0.07
15	27.984	1136	1143	*Trans*-verbenol	0.47 ± 0.03
16	29.753	1137	1175	Terpinen-4-ol	4.23 ± 0.28
17	30.204	1197	1185	Cymen-8-ol	1.72 ± 0.05
18	30.476	1198	1192	*α*-terpineol	0.53 ± 0.07
19	32.034	1120	-	NI	1.60 ± 0.10
20	35.380	1250	1256	Carvotanacetone	0.51 ± 0.01
21	36.173	1262	1290	Thymol	0.65 ± 0.02
22	38.469	1344	1348	*α*-Cubebene	1.00 ± 0.04
23	39.736	1375	1378	Copaene	4.37 ± 0.32
24	40.160	1382	1387	*β*-bourbonene	3.74 ± 0.45
25	40.371	1435	1474	*γ*-Muurolene	1.36 ± 0.07
26	41.765	1414	1417	*β*-Caryophyllene	6.71 ± 0.83
27	42.142	1454	1456	*α*-Humulene	0.96 ± 0.02
28	42.315	1464	1467	*cis*-Muurola-4 (14), 5-diene	1.05 ± 0.03
29	43.227	1485	1486	Germacrene D	1.05 ± 0.06
30	44.286	1502	1510	*β*-Bisabolene	1.16 ± 0.09
31	45.525	1512	1515	*γ*-Cadinene	9.41 ± 1.02
32	46.867	1515	1518	*α*-Alaskene	1.13 ± 0.32
33	46.238	1518	1520	*δ*-Cadinene	4.21 ± 0.62
34	47.483	1537	1538	*α*-Cadinene	0.44 ± 0.03
35	47.672	1557	1560	Germacrene –B-	1.31 ± 0.04
36	48.522	1576	1578	Spathulenol	0.66 ± 0.08
37	48.771	1587	1583	Caryophyllene oxide	3.54 ± 0.92
38	49.852	1595	1635	σ-Cadinol	1.08 ± 0.08
39	50.737	1615	-	NI	1.26 ± 0.15
40	51.003	1631	1642	*trans*-Muurulol	1.15 ± 0.06
41	51.217	1662	1660	*α*-Cadinol	0.45 ± 0.05
42	51.540	1681	1692	Acorenone	1.24 ± 0.12
43	52.800	1699	1685	Edusma-4 (15),7-diene-1-β-ol	1.35 ± 0.22
44	55.813	1720	-	NI	0.06 ± 0.02
45	56.232	1728	-	NI	0.16± 0.01
46	58.752	1774	1827	Neophytadiene	2 ± 0.31
47	59.060	1841	1845	Phytone	3 ± 0.73
48	59.914	1853	-	NI	0.06 ± 0.01
49	61.102	1886	-	NI	0.10 ± 0.02
50	62.561	1903	-	NI	0.04 ± 0.002
51	63.765	1934	-	NI	0.02 ± 0.001
52	64.131	1961	-	NI	0.06 ± 0.01
53	66.425	2005	-	NI	1.20 ± 0.24
54	68.153	2035	-	NI	0.02 ± 0.003
55	68.781	2045	2110	Phytol	1.3 ± 0.08
56	69.532	2059	2140	Osthole	1.46 ± 0.12
57	70.821	2130	-	NI	0.02 ± 0.005
58	73.075	2407	2400	Tetracosane	0.40 ± 0.02
59	76.931	2841	-	NI	0.08 ± 0.01
60	80.123	3230	-	NI	1.40 ± 0.11
61	83.807	3600	3600	Hexatriacontane	0.49 ± 0.03
				Hydrocarbon monoterpenes	26.01
				Oxygenated monoterpenes	9.15
				Hydrocarbon sesquiterpenes	37.9
				Oxygenated sesquiterpenes	9.47
				Other	8.65
				Unidentified compounds (%)	8.82
				Number identified	44
				Total identified (%)	91.18

^[a]^ RT = retention time; ^[b]^ RI_cal_= retention index relative to *n*-alkanes (C_8_–C_20_) using HP-5MS column; ^[c]^ RI_lit_ = mass spectral data retention index found in the literature; ^[d]^ compounds enumerated according to their RI; ^[e]^ relative concentration expressed as a percentage. ^[f]^ Not identified.

**Table 2:** Antioxidant activity of *T. boveana* extracts and EO.

	DPPH Test IC_50_^[a]^	ABTS Test IC_50_^[a]^	Reducing Power Test A_0.5_^[a]^	Phenanthroline Test A_0.5_^[a]^	SNP Test A_0.5_^[a]^
Essential oil	223.59 ± 1.01	593.33 ± 15.65	442.67 ± 14.85	746.25 ± 12.37	205.67 ± 0.88
Hydroalcoholic extract	64.16 ± 1.77	167.093 ± 3.53	33.02 ± 0.81	11.64 ± 2.88	44.31 ± 0.06
CHCl_3_ extract	131.68 ± 0.01	410.07 ± 8.07	174.41 ± 8.35	30.08 ± 1.83	176.5 ± 0.50
AcOEt extract	91.34 ± 0.65	315.777 ± 17.18	145.33 ± 6.75	18.67 ± 0.60	84.92 ± 3.79
*n*-BuOH extract	47.45 ± 4.82	9.17 ± 0.8	23.42 ± 0.5	7.40 ± 0.82	32.21 ± 0.73
BHA ^[b]^	6.14 ± 0.41	1.81 ± 0.10	7.99 ± 0.87	0.93 ± 0.07	73.47 ± 0.88
BHT ^[c]^	12.99 ± 0.41	1.29 ± 0.30	152.24 ± 2.43	2.24 ± 0.17	>200
*α*-Tocopherol	13.02 ± 5.17	7.59 ± 0.53	34.93 ± 2.38	5.78 ± 0.30	63.41 ± 4.39
Tannic acid	7.74 ± 0.19	1.01 ± 0.16	41.07 ± 2.36	-	-
Ascorbic acid	13.94 ± 2.81	1.74 ± 0.10	6.37 ± 0.42	8.30 ± 0.76	>200

^[a]^ IC_50_ and A_0.5_ values are expressed as means ± SD of three parallel measurements (*p* < 0.05) in (μg/mL); ^[b]^ BHA = butylated hydroxyanisole; ^[c]^ BHT = butylated hydroxytoluene.

**Table 3:** R^2^ linear correlation coefficient that shows the correlations between the tests of *T. boveanan*-BuOH extracts.

	DPPH Test	ABTS Test	Reducing Power Test	Phenanthroline Test	SNP Test
DPPH test	1				
ABTS test	0.986915566	1			
Reducing power test	0.929473080	0.960317460	1		
Phenanthroline test	0.790179245	0.861792735	0.842666085	1	
SNP test	0.830664209	0.907226194	0.915478964	0.948088307	1

**Table 4:** Quantification of total phenolic and flavonoid constituents in *T. boveana* extracts.

Extract	Total Phenolic Content (μg GAE/mg)	Total Flavonoid Content (μg QE/mg)
Hydroalcoholic extract	391.26 ± 2.8	120.31 ± 0.28
CHCl_3_ extract	303.85 ± 2.8	114.79 ± 0.22
AcOEt extract	119.44 ± 1.02	79.48 ± 0.83
*n*-BuOH extract	563.70 ± 3.40	124.79 ± 0.26

**Table 5:** Sun protection factor determination in *T. boveana* extracts and EO.

	SPF_1_	SPF_2_	SPF_3_	Mean ± SD
Essential oil	8.25	9.85	9.92	9.08 ± 1.18
Hydroalcoholic extract	46.10	46.07	46.13	46.10 ± 0.03
CHCl_3_ extract	41.28	41.58	40.98	41.28 ± 0.3
AcOEt extract	37.22	38.47	36.14	37.25 ± 1.25
n-BuOH extract	46.40	46.40	46.40	46.40 ± 0.00
Vichy sunscreen	44.11	44.33	44.22	44.22 ± 0.1
Nivea sunscreen	50.11	49.89	50.33	50.11 ± 0.22

**Table 6:** Minimum bacterial concentration (MBC) and minimum inhibitory concentration (MIC) values of essential oil and aerial part extracts of *T. boveana*.

Bacteria Strain	Essential Oil		Hydroalcoholic Extract		CHCl_3_ Extract		AcOEt Extract		*n*-BuOH Extract		Control ^[a]^	
	MIC ^[b]^ μg/mL	MBC ^[b]^ μg/mL	MIC	MBC	MIC	MBC	MIC	MBC	MIC	MBC	MIC	MBC
*Staphyloccocus aureus* (ATCC 25923)	1.5 ± 0.2	8 ± 0.6	6 ± 0.6	20 ± 0.2	3 ± 0.1	5 ± 0.3	3 ± 0.2	7 ± 0.9	6 ± 0.4	18 ± 2.4	2 ± 0.2	6 ± 0.4
*Micrococcus luteus* strain MM DSM 113600	1.5 ± 0.1	4 ± 0.3	3 ± 0.1	10 ± 0.2	1.5± 0.1	8 ± 0.6	24 ± 0.2	50 ± 5.2	3 ± 0.2	10 ± 1.2	2 ± 0.1	6 ± 0.3
*Pseudomonas aeruginosa* (ATCC 27853)	3 ± 0.3	5 ± 0.4	48 ± 2.51	80 ± 4.74	6 ± 0.9	20 ± 3.2	12 ± 1.25	36 ± 3.4	48 ± 4.5	90 ± 5.2	8 ± 0.3	14 ± 1.85
*Escherichia coli* (ATCC 25922)	3 ± 0.6	8 ± 0.7	12 ± 1.2	36 ± 4.2	6 ± 0.8	17 ± 0.2	24 ± 2.23	72 ± 4.2	6 ± 0.4	10 ± 0.4	15 ± 3.1	23 ± 4.15

^[a]^ Positive control (Chloramphenicol/Gentamycin). ^[b]^ Values are expressed as means ± SD of three measurements (*p* < 0.05) in (μg/mL).

**Table 7:** List of the major constituents of *T. boveana* EO with their docking scores, interaction types between interacting residues and bond distances towards the BuChE enzyme (PDE 4bds).

Name	Docking Score kcal/mol	Interaction Types	Interacting Residues	Interaction Distance/Å
*γ*-Cadinene	−7.6	Pi–alkyl	HSD438	4.90
		Pi–sigma, Pi–alkyl	TRP82	3.58, 5.14, 4.12
*β*-caryophyllene	−7.5	Pi–sigma	TRP82	3.76, 3.86
		Pi–alkyl	TYR332	5.30
		Pi–alkyl	ALA328	4.30
Limonene	−5.9	Pi–alkyl, alkyl	TRP82	4.21, 4.47
		Pi–alkyl, alkyl	HSD438	5.17, 5.48
		Pi–alkyl, alkyl	MET437	5.48
		Pi–alkyl, alkyl	TYR440	5.17
		Pi–alkyl, alkyl	ALA328	4.26
*p*-Cymene	−6.1	Pi–sigma, Pi–alkyl	TRP82	4.15, 5.32
		Pi–sigma	HSD438	5.73
		Pi–alkyl	ALA328	4.37
Copaene	−7.3	Pi–sigma, Pi–alkyl	TRP82	3.77, 5.20, 4.77
		Pi–alkyl	TYR332	5.29
		Pi–alkyl	HSD438	5.27
Terpinen-4-ol	−5.9	Pi–alkyl	TRP82	3.88, 5.22
		Pi–alkyl	ALA328	4.38
*δ*-Cadinene	−7.6	Pi–alkyl	HSD438	4.72
		Pi–sigma, Pi–alkyl	TRP82	3.74, 4.60, 4.52, 4.16
*γ*-Terpinene	−6.1	Pi–sigma, Pi–alkyl	TRP82	4.16, 5.26
		Pi–sigma	HSD438	5.74
		Pi–alkyl	ALA328	4.34

**Table 8:** List of the major constituents of *T. boveana* EO with their docking scores, interaction types between interacting residues and bond distances toward *α*-amylase enzyme (PDE 4gqr).

Name	Docking Score kcal/mol	Interaction Nature	Interacting Residues	Interaction Distance/Å
*γ*-Cadinene	−7.3	Pi–alkyl	TRP58	5.08, 5.27
		Pi–alkyl	TRP59	4.67, 4.52
		Pi–alkyl	HSD305	5.13
*β*-caryophyllene	−6.9	-	-	-
Limonene	−5.8	Pi–sigma, Pi–alkyl	TYR62	3.94, 4.61
		Pi–alkyl	LEU165	4.77
		Pi–alkyl	HSE299	4.84
*p*-Cymene	−5.9	Pi–sigma, Pi–alkyl	TYR62	3.87, 4.47
		Pi–alkyl	HSE299	4.74
Copaene	−7.3	Pi–sigma, Pi–alkyl	TYR62	3.90, 5.25
		Pi–alkyl	ALA198	4.73
		Pi–alkyl	LEU165	4.98
		Pi–alkyl	LEU162	5.25, 5.27
Terpinen-4-ol	−5.1	Pi–sigma, Pi–alkyl	TYR62	3.84, 4.42
		Pi–alkyl	HSE299	4.90
*δ*-Cadinene	−7.0	Pi–alkyl	TRP59	4.98
		Pi–alkyl	TRP58	5.20
		Pi–alkyl	HSE299	4.58
		Pi–sigma, Pi–alkyl	TYR62	3.86, 4.97
*γ*-Terpinene	−5.9	Pi–sigma, Pi–alkyl	TYR62	3.90, 4.46
		Pi–alkyl	HSE299	4.70

## Data Availability

The data are contained in the article.

## Supplementary Materials

The following supporting information can be downloaded at: https://www.mdpi.com/article/10.3390/plants14162497/s1 Table S1: The cartesian coordinates of the eight main components of the EO of Tamarix boveana computed at B3LYP/6-311G(d,p) level in the gas phase.

## Abbreviations

The following abbreviations are used in this manuscript:

EO	Essential Oil
GC-MS	Gas Chromatography–Mass Spectrometry
GC-FID	Gas Chromatography–Flame Ionization Detector
CHCl_3_	Chloroform
AcOEt	Ethyl Acetate
*n*-BuOH	*n*-Butanol
MIC	Minimum Inhibitory Concentration
MIB	Minimum Bactericidal Concentration
DFT	Density Functional Theory
BuChE	Butyryl Cholinesterase Enzyme
AD	Alzheimer’s Disease
DM	Diabetes Mellitus
DPPH	2,2-Diphenyl-1-picrylhydrazyl
FRAP	Ferric Reducing Antioxidant Power
IC_50_	Half Maximal Inhibitory Concentration
RT	Retention Time
RI	Retention Index
Relat.	Conc. Relative Concentration Expressed as a Percentage
TPC	Total Phenolic Content
AGE	Gallic Acid Equivalents
QE	Quercetin Equivalents
TFC	Total Flavonoids Content
ROS	Reactive Oxygen Species
SNP	Silver Nanoparticles
ABTS	2,2′-Azino-Bis(3-ethylbenzoThiazoline-6-Sulfonic Acid)
BHA	Butylated HydroxyAnisole
BHT	Butylated HydroxyToluene
SPF	Sun Protection Factor
HOMO	Highest-Occupied Molecular Orbital
LUMO	Lowest-Occupied Molecular Orbital
ESP	Electrostatic Potential Maps
