# Supplementary material for: Chemical Constituents, In Silico Studies and In Vitro Antioxidant, Enzyme Inhibitory and Antibacterial Activities of the Algerian Tamarix boveana Essential Oil and Extracts
Source: Plants (Basel). 2025 Aug 11;14(16):2497. doi: 10.3390/plants14162497 (PMC12389186; doi:10.3390/plants14162497)
Supplement: Supplementary file 1 [file plants-14-02497-s001.zip › plants-3760845-supplementary.pdf]

Supplementary Materials

Table S1. The cartesian coordinates of the eight main components of the EO of *Tamarix boveana* computed at B3LYP/6-311G(d,p) level in the gas phase.

γ-cadinene			
C	-0.11261720	-0.12507965	0.26782087
C	1.29048563	-0.20498068	-0.40610252
C	-0.91360525	1.08815523	-0.28675441
C	2.03813328	1.12564792	-0.18715715
C	2.13514566	-1.44345021	0.00924532
C	-2.35060784	1.06098787	0.23997897
C	-0.14423524	2.36913367	-0.04724310
C	1.23200102	2.34688090	-0.66484863
C	-0.92799249	-1.39410186	0.14687312
C	-3.07949336	-0.20016982	-0.23389093
C	-2.24301839	-1.45055819	-0.08736278
C	3.43427648	-1.55429854	-0.80653229
C	2.43634575	-1.52719829	1.51418187
C	-0.59234315	3.40562072	0.66172681
C	-2.97883991	-2.75801071	-0.21755504
H	0.05814352	0.06979091	1.33780830
H	1.11318187	-0.31092406	-1.48682700
H	-0.96309867	0.94871397	-1.37875289
H	2.99951226	1.10906448	-0.70721392
H	2.25709311	1.24996030	0.87940525
H	1.54251945	-2.32604917	-0.25199469
H	-2.89316889	1.95114039	-0.09147224
H	-2.33733250	1.08517510	1.33579043
H	1.13258106	2.29786252	-1.75819455
H	1.77112212	3.27014971	-0.43505936
H	-0.39936170	-2.33324086	0.28040240
H	-3.38113258	-0.09168967	-1.28576466
H	-4.01560130	-0.32468847	0.32434844
H	4.15995953	-0.78345454	-0.53254415
H	3.91160758	-2.52345686	-0.63415022
H	3.24039791	-1.46650809	-1.88012696
H	3.05491451	-0.69028893	1.85100486
H	2.98474561	-2.44660685	1.73963189
H	1.52544475	-1.53353100	2.11766825
H	-1.57445352	3.41937782	1.11861410

H	0.02135541	4.28876702	0.80729377
H	-3.49243964	-2.82397740	-1.18436610
H	-3.75260580	-2.85377788	0.55350656
H	-2.30519407	-3.61327866	-0.13328823
β-caryophyllene			
C	-2.46051541	-0.06796508	0.05164640
C	-0.97368006	0.43907618	0.14460619
C	-0.50105609	-0.97492478	-0.37389141
C	-1.90272903	-1.51977964	0.02137315
C	-0.58680677	1.73770547	-0.57855477
C	-3.35108843	0.29169133	1.23917198
C	-3.16299542	0.29088259	-1.26419772
C	0.74080460	-1.68145269	0.14789540
C	0.70576670	2.41700201	-0.07109609
C	1.91274136	-1.86939472	-0.80835036
C	1.86339718	1.44184647	0.09280722
C	2.92573591	-0.68485703	-0.80055888
C	0.81611852	-2.15562851	1.39405328
C	2.16547189	0.60200906	-0.90201315
C	2.51016078	1.42949013	1.45384206
H	-0.71342082	0.51814028	1.20647307
H	-0.43759565	-0.92796582	-1.46495678
H	-2.37012355	-2.22237999	-0.67388413
H	-1.89701016	-1.97001780	1.01621081
H	-1.40813215	2.45685519	-0.46951168
H	-0.50639583	1.54342388	-1.65312997
H	-3.55136049	1.36858342	1.27181930
H	-4.31729556	-0.22110914	1.17805372
H	-2.88194335	0.00979065	2.18643875
H	-2.55802815	0.04005335	-2.13967579
H	-4.10420222	-0.26231988	-1.34570610
H	-3.40180755	1.35727146	-1.31829597
H	0.49897218	2.88041038	0.89960284
H	0.97227572	3.24133611	-0.74388997
H	2.44870285	-2.79036603	-0.55970280
H	1.52702630	-1.99536945	-1.82641729
H	3.62443558	-0.81538242	-1.63520143
H	3.51087559	-0.73037450	0.11983000
H	0.00999213	-2.04207131	2.11063660
H	1.70014605	-2.67651256	1.74753388
H	1.63619071	0.73313900	-1.84534513
H	1.79630616	1.11530276	2.22423516
H	2.83599782	2.44171959	1.72117186

H	3.37844012	0.77363823	1.51331129
limonene			
C	0.71642667	0.01260578	-0.26641813
C	0.04200262	-0.99082247	0.69641670
C	-0.02640234	1.35388902	-0.17936506
C	-1.42034828	-1.23742948	0.30451682
C	-2.16843950	0.03082862	-0.04340403
C	-1.52373175	1.18420511	-0.24798569
C	2.21985315	0.08297898	-0.04890452
C	-3.66540352	-0.08869039	-0.15182589
C	3.00749668	-1.04877120	-0.66486208
C	2.83321688	1.05354538	0.63343997
H	0.56200654	-0.37371973	-1.28472414
H	0.09029802	-0.57836353	1.71074540
H	0.58516033	-1.93980852	0.71637385
H	0.30888787	2.01528282	-0.98624903
H	0.23059817	1.87184612	0.75369929
H	-1.94097803	-1.75197587	1.12204757
H	-1.47110439	-1.92674959	-0.55108806
H	-2.10187163	2.07626020	-0.48025699
H	-3.94646209	-0.84387912	-0.89586068
H	-4.12682647	0.85846273	-0.43951318
H	-4.10579604	-0.40837073	0.80007980
H	2.64481149	-2.02429993	-0.32391501
H	2.90437118	-1.03967635	-1.75623092
H	4.06950894	-0.97938904	-0.42235187
H	3.90902805	1.04318364	0.77389518
H	2.30034448	1.88716074	1.07370019
p-cymene			
C	2.13739774	0.38030442	0.00665745
C	0.62424205	0.21817344	0.00195485
C	2.78340363	-0.19498591	-1.26711146
C	2.77787589	-0.22156797	1.27088418
C	0.01965205	-1.04580940	-0.00975732
C	-0.21637529	1.33330117	0.00536611
C	-2.20594025	-0.06345739	-0.00873046
C	-1.36466851	-1.18298745	-0.01574026
C	-1.60496067	1.19656843	-0.00069226
C	-3.70753562	-0.22059794	0.00989125
H	2.34023407	1.45752572	0.01833561
H	2.63379792	-1.27678204	-1.33446065
H	3.86164775	-0.00810689	-1.27068247
H	2.35619817	0.25718517	-2.16582697

H	2.34739409	0.21237279	2.17699557
H	3.85623282	-0.03572922	1.28255312
H	2.62717987	-1.30440041	1.31537606
H	0.63490071	-1.93969584	-0.01700421
H	0.21874611	2.32816902	0.01018305
H	-1.80030641	-2.17776629	-0.02764297
H	-2.22747655	2.08609383	-0.00054729
H	-4.20818353	0.71227698	-0.25827035
H	-4.03673674	-0.99348758	-0.69011574
H	-4.06217436	-0.51130363	1.00477480
copaene			
C	-1.04530580	1.21650645	-0.02857495
C	0.21258187	-0.51247682	0.10155418
C	-0.48807944	0.38018550	1.18059664
C	-1.00872505	-0.15150367	-0.80512102
C	1.54150684	0.05907833	-0.42946845
C	0.05635570	2.13741627	-0.59614294
C	1.47574021	1.59972474	-0.34901838
C	-1.59506652	-0.42162786	1.88482570
C	-2.17808592	-1.04063481	-0.42649103
C	-2.35686053	1.97900424	0.10458585
C	2.78833950	-0.51650482	0.29352344
C	-2.43581439	-1.17201757	0.87848603
C	-2.95610470	-1.71757747	-1.51749175
C	2.94186273	-2.03150048	0.08192733
C	4.08545535	0.18485748	-0.14343986
H	0.28975970	-1.57300459	0.34344618
H	0.13778277	0.90260985	1.90995190
H	-0.84453514	-0.10683546	-1.88666949
H	1.62215942	-0.23586151	-1.48690854
H	-0.03254957	3.13394268	-0.14878597
H	-0.12672391	2.26766541	-1.67021201
H	2.18157740	2.06288425	-1.04260115
H	1.79329295	1.90392824	0.65468419
H	-2.22727189	0.24801910	2.48446359
H	-1.14298500	-1.12028458	2.60174253
H	-3.18425437	1.33648856	0.40801642
H	-2.25825439	2.78167884	0.84355504
H	-2.62700610	2.44560173	-0.84947321
H	2.65565034	-0.33522726	1.36997389
H	-3.24851487	-1.79861444	1.23614377
H	-3.76096220	-2.33659778	-1.11454626
H	-2.30583020	-2.35558165	-2.12796717

H	-3.39896626	-0.98142965	-2.19907079
H	3.06804132	-2.26271619	-0.98165959
H	3.82588009	-2.40494295	0.60685578
H	2.08337466	-2.59858179	0.44610795
H	4.23838713	0.08044083	-1.22366017
H	4.95044863	-0.26161913	0.35541296
H	4.08470049	1.25046057	0.09269544
terpinen-4-ol			
O	0.62645829	-0.12727794	1.43216649
C	0.61007000	0.00077971	-0.00305471
C	2.09730072	0.01457500	-0.45118356
C	-0.16716284	-1.18168735	-0.61848848
C	-0.11941571	1.30259474	-0.39340778
C	-1.60711862	-1.25675839	-0.09529359
C	-2.30423362	0.08436803	-0.06248829
C	-1.61576782	1.22154927	-0.20600447
C	2.84311151	1.26786036	0.03628374
C	2.85792491	-1.24237442	0.00444971
C	-3.79407634	0.06121913	0.15116767
H	2.08525365	0.02856299	-1.54904623
H	0.34440530	-2.12019560	-0.40078582
H	-0.17952100	-1.06035349	-1.70785574
H	0.11182409	1.55790136	-1.43667753
H	0.27441369	2.12366364	0.21252565
H	-1.61486146	-1.69075469	0.91407454
H	-2.18831324	-1.95356421	-0.71079587
H	-2.15138674	2.16821356	-0.18248038
H	2.78881796	1.34112500	1.12503253
H	3.89735291	1.21395495	-0.24858404
H	2.43842096	2.18872333	-0.38920052
H	2.82635944	-1.33301075	1.09218500
H	3.90530213	-1.17818070	-0.30316097
H	2.44971421	-2.15998117	-0.42395306
H	-0.24247685	0.13095714	1.75723124
H	-4.21207948	1.06864258	0.20349865
H	-4.05110003	-0.46327082	1.07920230
H	-4.29758492	-0.47696621	-0.66042303
δ-cadinene			
C	1.34414818	-0.02407784	-0.29425660
C	0.02084224	-0.28561988	0.47374734
C	1.76016744	1.44103362	-0.11126328
C	-0.97910629	0.86525778	0.40382184
C	2.47093957	-1.03249850	0.06941023

C	0.68896473	2.38408804	-0.65240430
C	-0.69724451	2.05988633	-0.13797441
C	-2.32004398	0.46669640	0.97248639
C	-0.64378717	-1.56958731	-0.00039394
C	-2.95201810	-0.66409550	0.14509838
C	-1.95539063	-1.75222939	-0.17730261
C	3.56561358	-1.06755364	-1.00901011
C	3.09261286	-0.80381536	1.45689241
C	-1.68063922	3.19363671	-0.29772574
C	-2.52382222	-3.04311272	-0.70474649
H	1.12155701	-0.17006261	-1.36057105
H	0.28134222	-0.42233914	1.53688090
H	2.71487418	1.63825578	-0.60809497
H	1.90834792	1.64976713	0.95407568
H	2.01831992	-2.03036193	0.08447534
H	0.68351856	2.36113519	-1.75211463
H	0.93512178	3.41933393	-0.38435753
H	-2.16422938	0.09614068	1.99439552
H	-3.00709784	1.30908156	1.05017135
H	0.02293647	-2.39757338	-0.22753083
H	-3.36509507	-0.26105592	-0.78992761
H	-3.80385688	-1.09600304	0.68606588
H	4.09466607	-0.11239545	-1.08246880
H	4.31148536	-1.83467327	-0.78069860
H	3.14531223	-1.29302475	-1.99352224
H	3.66239196	0.12896089	1.49586914
H	3.78240004	-1.61719892	1.70028863
H	2.33891780	-0.76809676	2.24805809
H	-1.66483030	3.56025766	-1.33097266
H	-1.40018388	4.04413843	0.33583378
H	-2.70902187	2.92456661	-0.06103497
H	-3.11183500	-2.86955436	-1.61413493
H	-3.20319254	-3.50261288	0.02310689
H	-1.73926761	-3.76473800	-0.94206699
γ-terpinene			
C	2.17908880	0.35150869	-0.00018950
C	0.66336313	0.16287868	-0.00015471
C	-0.16490607	1.42477382	-0.00047196
C	-1.40021333	-1.27257864	0.00030937
C	0.08570444	-1.03882141	0.00019735
C	-2.22535622	-0.00881396	0.00000769
C	-1.64958627	1.19397432	-0.00034246
C	2.83571033	-0.21950306	1.26934253

C	2.83587382	-0.22095443	-1.26897610
C	-3.71874532	-0.19305079	0.00013293
H	2.36551333	1.43251718	-0.00079738
H	0.11600658	2.04064081	-0.86959712
H	0.11608382	2.04113478	0.86827724
H	-1.68026989	-1.88887799	-0.86860461
H	-1.68020743	-1.88842241	0.86956740
H	0.70523181	-1.93293141	0.00042269
H	-2.27719101	2.08267425	-0.00055545
H	2.71256243	-1.30504797	1.32045177
H	2.39087940	0.20768628	2.17197389
H	3.90843011	-0.00262871	1.28367091
H	2.39141062	0.20547795	-2.17214872
H	2.71243252	-1.30651292	-1.31904988
H	3.90866245	-0.00440525	-1.28325016
H	-4.04603068	-0.76153155	-0.87868971
H	-4.24316172	0.76473673	-0.00013820
H	-4.04595225	-0.76098913	0.87933667
